# Nursing Students' Perceptions, Knowledge, and Preventive Behaviors Toward COVID-19: A Multi-University Study

**DOI:** 10.3389/fpubh.2020.573390

**Published:** 2020-12-23

**Authors:** Hamdan Mohammad Albaqawi, Nahed Alquwez, Ejercito Balay-odao, Junel Bryan Bajet, Hawa Alabdulaziz, Fatmah Alsolami, Regie B. Tumala, Abdalkarem F. Alsharari, Hanan M. M. Tork, Ebaa Marwan Felemban, Jonas Preposi Cruz

**Affiliations:** ^1^College of Nursing, University of Hail, Hail, Saudi Arabia; ^2^Nursing Department, College of Applied Medical Sciences, Shaqra University, Al Dawadmi, Saudi Arabia; ^3^Faculty of Nursing, King Abdulaziz University, Jeddah, Saudi Arabia; ^4^Faculty of Nursing, Umm Al-Qura University, Makkah, Saudi Arabia; ^5^College of Nursing, King Saud University, Riyadh, Saudi Arabia; ^6^Nursing Department, College of Applied Medical Sciences, Jouf University, Sakakah, Saudi Arabia; ^7^College of Nursing, Qassim University, Qassim, Saudi Arabia; ^8^Faculty of Nursing, Taif University, Taif, Saudi Arabia

**Keywords:** COVID-19, knowledge, nursing–education, perception, preventive behavior, Saudi Arabia (KSA)

## Abstract

**Background:** Knowledge, perception, and preventive behavior should be considered in the planning of effective educational interventions for the coronavirus disease of 2019 (COVID-19) pandemic and in increasing awareness about the health risks brought about by this disease. This research aimed to assess knowledge, perceptions, and preventive behavior toward the COVID-19 infection among student nurses.

**Methods:** The study has quantitative, descriptive, and cross-sectional design. A convenience sample of 1,226 student nurses from seven universities in Saudi Arabia was surveyed from March 22 to April 4, 2020. A four-part online survey on demographic characteristics, perceptions, knowledge, and preventive behavior of Saudi student nurses was carried out.

**Results:** Nearly all students were aware of the outbreak (99.2%), and most of them received information on COVID-19 primarily from social media (71.0%). Over three-fourths of the students were confident that the government (89.1%) and Ministry of Health (MOH) (86.5%) were doing a good job responding to the COVID-19 outbreak in the country. The overall average score in the knowledge questionnaire was 9.85 (SD = 1.62, range = 0–12), which is equivalent to 82.1%. The majority of the students always performed most of the preventive behavior identified in the survey, except “washing hands with soap and water for at least 20 s after blowing my nose, coughing, or sneezing” (39.2%) and “daily cleaning and disinfecting frequently touched surfaces” (41.6%). Being female, being in the fourth year, and gaining good perceived knowledge were associated with high actual COVID-19 knowledge. University, gender, age, academic level, and perceived COVID-19 knowledge were the associated factors.

**Conclusions:** The findings of this study have provided baseline information on the current state of Saudi nursing students' perceptions, knowledge, and preventive behavior toward COVID-19 as the crisis is happening. The findings revealed some areas that should be focused on by nursing education, as well as health agencies, to ensure that the students have adequate knowledge and correct preventive behavior.

## Introduction

The fight against coronavirus disease of 2019 (COVID-19) remains ongoing in Saudi Arabia and around the world. To date, there are more than 41.5 million confirmed cases globally and more than 340,000 confirmed cases in Saudi Arabia (1). The COVID-19 pandemic has immensely affected all aspects of society (2). At present, a vaccine and medicine against COVID-19 have yet to be developed, and the prevention and control of this disease are the major challenges that every country faces.

Since the outbreak of the disease, different governments around the world have been implementing measures to contain and prevent the transmission of COVID-19. The World Health Organization published COVID-19 guidelines and protocols, which were adopted by the ministries of health of different countries (3). These protocols include information on signs and symptoms and prevention of and protective measures against COVID-19. The Centers for Disease Control and Prevention reiterated that everyone should protect themselves and others to prevent the spread of the disease; such protection includes proper hand hygiene, proper distancing, use of mask, proper etiquette when coughing and sneezing, and isolation and decontamination of surfaces (4). The success of the measures implemented is based on the people's adherence to prevention controls, which is largely influenced by knowledge, perception, and preventive behavior against COVID-19 (5). In the US, 47% of the surveyed population are willing to engage in preventive behavior (e.g., hand hygiene by using soap, water, and disinfectants, such as hand sanitizers) (6). However, adapting these preventive and control behavior requires adequate knowledge, right perception, and positive attitudes, as proposed by the Knowledge–Attitudes–Behavior (KAB) model (7, 8).

The KAB model is a vital health education theoretical model that explains the part of knowledge in behavioral changes and emphasizes that changes in behavior are a product of knowledge and attitudes (7). This model proposes that human health behavior can be modified through three continuous processes of change, namely, gaining of knowledge, formation of beliefs, and development of behavior (8, 9). KAB emphasizes that the knowledge of a person can directly affect attitude and indirectly affect behavior through attitude (8). In the present study, knowledge and information received by student nurses about COVID-19 may affect their attitudes to it, and attitude may affect their behavior or actions. Providing students with health information and knowledge through various sources and means is intended to enhance the health related behavior, attitudes, and practices of student nurses with regard to the prevention and control of COVID-19. However, the negative perception of COVID-19 information and misinformation can lead to poor knowledge and practice behavior (10). Thus, the most essential method to stop the spread of the COVID-19 pandemic is to develop and adopt appropriate preventive behavior, which can be achieved by becoming well-versed in this disease (11).

However, whether student nurses possess adequate knowledge, positive perceptions, and appropriate preventive behavior in relation to COVID-19 remains unexplored. Being components of the nursing curriculum, preventive measures are no longer new to nursing students, but experiencing a pandemic is new to everyone. Thus, the knowledge, perception, and preventive behavior of nursing students may be affected. This notion has been supported by some studies on previous health crises brought about by infectious diseases, such as the Middle East Respiratory Syndrome (MERS)-Cov. For instance, in the study Choi and Kim, student nurses reported low-risk perception of and poor preventive attitudes toward MERS (12). However, to the best of our knowledge, no research on this issue has been conducted among student nurses in Saudi Arabia. In crises, such as the current one, student nurses' knowledge, perception, and preventive behavior should be considered in the planning of effective educational interventions for COVID-19 and in increasing awareness of the health risks brought about by this disease. Therefore, this research aimed to assess the perceptions, knowledge, and preventive behavior of nursing students toward the COVID-19.

## Materials and Methods

This quantitative, descriptive, and cross-sectional research surveyed nursing students from seven government universities in Saudi Arabia. University A is situated in the northern region. Universities C–E are in the central area. Universities B, F, and G are in the western region. A convenience sample of 1,226 student nurses who met the following criteria was surveyed in this study: (1) registered in the BSN program during the conduct of the study; (2) sophomores, juniors, seniors, or interns; (3) both sexes; and (4) living as a Saudi national.

An online survey in the Arabic language was used for data collection. The online survey had four parts. Part 1 contained the study information. Part 2 asked for the following information: (1) age, (2) sex, (3) university, (4) year level, (5) awareness of the novel coronavirus outbreak in the country, (6) awareness of infected persons in their immediate society, (7) perceived knowledge of COVID-19, (8) perceived knowledge of the prevention and control of COVID-19, (9) sources of COVID-19 information, (10) learning COVID-19 in any of the nursing courses and activities, (11) family members working in hospitals, (12) confidence on how the government responds to the pandemic, and (13) confidence about how Ministry of Health (MOH) responds to the pandemic.

Part 3 comprises an adapted questionnaire from Zhong et al. (13) that measures the COVID-19 knowledge. The questionnaire was developed based on the “guidelines for clinical and community management of COVID-19 by the National Health Commission” of China. The questionnaire had 12 questions covering three aspects: (a) knowledge of the clinical manifestations (four items), (b) knowledge of the mode of transmission (three items), and (c) knowledge of the prevention and control (five items). The items were answered with “true, false, or I don't know” response options. Correct answers were scored as 1, whereas incorrect answers or “I don't know” answers were scored as 0. We added the correct answers to determine the knowledge of the students. High scores indicated superior COVID-19 knowledge. The questionnaire had good reliability, with Cronbach's alpha of 0.71 (12).

Part 4 included statements pertaining to prevention practices for COVID-19. This part was developed by researchers based on CDC (14). Initially, the questionnaire comprised 14 questions on the recommended steps for protecting oneself and others. A panel of nine experts on infection control and prevention evaluated the content validity of the scale. The panel was composed of (1) five assistant professors in nursing who had PhD with specialization in medical–surgical nursing, (2) two medical doctors who specialize in infectious diseases, and (3) two nurses who work as infection prevention and control nurses in a hospital. Nine items had an item-level content validity index of 1, four items had 0.89, and one had 0.56. The researchers decided to exclude the last item, leaving 13 items for the final scale. The scale-level content validity index of the 13 items was 0.97. The students were asked how often they perform the activities in the past week by selecting from three options: always, sometimes, and never. The responses of the students were coded as 2 for always, 1 for sometimes, and 0 for never. Mean scores were calculated with high means, indicating excellent preventive behaviors. The scale was distributed to 50 Saudi nursing students for pilot testing. The analysis revealed a Cronbach's alpha of 0.976.

This research was reviewed and approved by the Nursing Research Ethical Committee of King Abdulaziz University. The participants were recruited online by each collaborator at their respective universities. An online survey link was forwarded to the students through university e-mails, phone numbers, and social groups, which are common for nursing students in the country. A research information sheet, which contains a brief background of the study, importance of the investigation, objectives of the study, needed participation, rights of the respondents, and voluntary nature of participation, was included in the first part of the online survey. The students were asked to proceed with the online survey if they agree to join. Students who completed the survey were deemed to have agreed to participate in the study. We did not collect identification information from the students to ensure privacy and confidentiality throughout the study. A completed survey was automatically registered online. The data collection was from March 22 to April 4, 2020.

The researchers used SPSS version 22.0 for data analyses. Descriptive statistics were used in analyzing and describing different variables (e.g., demographics, perceptions, knowledge, and preventive behavior). Pearson's product correlation, *t*-test, and one-way ANOVA were performed for the examination of demographic variables associated with the nursing students' COVID-19 knowledge and preventive behavior. Tukey's honestly significant difference (HSD) test was used as *post hoc* analysis when ANOVA revealed significant relationships.

## Results

The highest number of respondents was obtained from University B (19.7%), whereas the lowest was obtained from University F (7.3%). The students were 18–45 years old (M = 21.62, SD = 2.06). Over two-thirds (71.6%) of the students were female, and over one-third were sophomores (38.4%). Additionally, 25.3, 21.6, and 14.7% of the students were juniors, seniors, and interns, respectively (Table 1).

**Table 1:** The demographic characteristic and perceptions related to COVID-19 among the Saudi nursing students (*n* = 1,226).

**Demographic variables and COVID-19 perceptions**	**Mean (SD)**	**Range**
Age in years	21.62 (2.06)	18–45
University	*N*	%
University A	165	13.5
University B	242	19.7
University C	170	13.9
University D	156	12.7
University E	201	16.4
University F	90	7.3
University G	202	16.5
Gender		
Male	348	28.4
Female	878	71.6
Year of study		
2nd year	471	38.4
3rd year	310	25.3
4th year	265	21.6
Internship year	180	14.7
Awareness of COVID-19 outbreak		
No	10	0.8
Yes	1,216	99.2
Know people in their community infected with COVID-19		
No	1,136	92.7
Yes, confirmed	45	3.7
Yes, but not yet confirmed	45	3.7
Primary source of COVID-19 information		
Television	81	6.6
Social media	870	71.0
Newspaper	58	4.7
Friends	11	0.9
Relatives working in the medical field	129	10.5
Relatives not working in the medical field	2	0.2
University	75	6.1
Learned about coronavirus in any nursing course		
No	884	72.1
Yes	342	27.9
Family member working in a healthcare facility		
No	553	45.1
Yes	673	54.9
Confidence that government is doing a good job		
responding to the COVID-19 outbreak		
Not at all confident	15	1.2
Not too confident	22	1.8
Somewhat confident	97	7.9
Very confident	1,092	89.1
Confidence that the Ministry of Health is doing a good job		
responding to the COVID-19 outbreak		
Not at all confident	13	1.1
Not too confident	29	2.4
Somewhat confident	124	10.1
Very confident	1,060	86.5
	Mean (SD)	Range
Perceived knowledge on COVID-19^a^	7.85 (1.87)	0–10
Perceived knowledge on COVID-19 prevention^a^	8.51 (1.81)	0–10

a *Possible range of scores = 0–10*.

### Perceptions Related to COVID-19

Table 1 shows the students' perception of information on COVID-19. Nearly all students were aware of the COVID-19 outbreak (99.2%). A vast majority of the students did not know any person within their immediate community with a confirmed COVID-19 infection (92.7%). However, 45 students (3.7%) knew someone with a confirmed case in their communities, and another 24 students (3.7%) knew someone with suspected COVID-19 infection in their communities. The majority of the students had received their information on COVID-19 primarily from social media (71.0%), relatives working in the medical field (10.5%), television (6.6%), university (6.1%), newspaper (4.7%), and friends (0.2%). Moreover, the majority of the students reported that they had not learned of the coronavirus from any of their nursing courses (72.1%) and had a family member working in any healthcare facility (54.9%). Over three-fourths of the students were confident that the government (89.1%) and MOH (86.5%) were doing a good job responding to the COVID-19 outbreak in the country. The mean scores in the perceived knowledge of COVID-19 and perceived knowledge of the prevention of COVID-19 were 7.85 (SD = 1.87) and 8.51 (SD = 1.81), respectively, on a scale of 0–10.

### Knowledge of COVID-19 and Associated Factors

The overall average score in the knowledge questionnaire was 9.85 (SD = 1.62, range = 0–12), which is equivalent to 82.1%. For the conceptual subscales, the students received the highest percentage of 87.6% in “prevention and control” (M = 4.38, SD = 0.79, range = 0–5), followed by 81.0% in “clinical presentation” (M = 3.24, SD = 0.84, range = 0–4) and 74.3% in “transmission route” (M = 2.23, SD = 0.73, range = 0–3). Table 2 indicates that the highest percentage of correct responses was recorded in the statement on isolating someone exposed to COVID-19 for 14 days (98.5%), followed by isolating and treating COVID-19 patients for the prevention of the disease's spread (98.0%), avoiding crowded places for the prevention of the spread (95.5%), transmission of the virus through respiratory droplets (92.0%), common clinical manifestation of COVID-19 (91.6%), and significance of treating the symptoms of the disease in helping patients recover (90.5%). Most students were not knowledgeable about that possibility that eating or being in contact with wild animals can cause infection (% of correct response = 47.1%).

**Table 2:** Results of the descriptive analyses on the Saudi nursing students' knowledge regarding COVID-19 (*n* = 1226).

**Survey items**	**Students who answered correctly *n* (%)**	**Mean score (SD)**
Clinical presentation (four items)^a^		3.24 (0.84)
The main clinical symptoms of COVID-19 are fever, fatigue, dry cough, shortness of breath, diarrhea, and myalgia.	1,123 (91.6)	
Unlike the common cold, stuffy nose, runny nose, and sneezing are less common in persons infected with the COVID-19 virus.	857 (69.9)	
There currently is no effective cure for COVID-2019, but early symptomatic and supportive treatment can help most patients recover from the infection.	1,109 (90.5)	
Not all persons with COVID-2019 will develop to severe cases. Only those who are elderly, have chronic illnesses, and are obese are more likely to be severe cases.	878 (71.6)	
Transmission route (three items)^b^		2.23 (0.73)
Eating or contacting wild animals would result in the infection by the COVID-19 virus.	578 (47.1)	
Persons with COVID-2019 cannot infect the virus to others when a fever is not present.	1025 (83.6)	
The COVID-19 virus spreads via respiratory droplets of infected individuals.	1,128 (92.0)	
Prevention and control (five items)^c^		4.38 (0.79)
Ordinary residents can wear general medical masks to prevent the infection by the COVID-19 virus.	694 (56.6)	
It is not necessary for children and young adults to take measures to prevent the infection by the COVID-19 virus.	1,100 (89.7)	
To prevent the infection by COVID-19, individuals should avoid going to crowded places such as train stations and avoid taking public transportations.	1,171 (95.5)	
Isolation and treatment of people who are infected with the COVID-19 virus are effective ways to reduce the spread of the virus.	1,201 (98.0)	
People who have contact with someone infected with the COVID-19 virus should be immediately isolated in a proper place. In general, the observation period is 14 days.	1,208 (98.5)	

a *Possible range of scores = 0–4*,

b *Possible range of scores = 0–3*,

c *Possible range of scores = 0–5*.

Pearson's correlations revealed positive correlations between COVID-19 knowledge and perceived knowledge of COVID-19 (*r* = 0.23, *p* < 0.001) and perceived knowledge on COVID-19 prevention (*r* = 0.27, *p* < 0.001). One-way ANOVA showed significant differences on COVID-19 knowledge in terms of university (*F* = 6.19, *p* < 0.001) and academic levels (*F* = 4.01, *p* = 0.008). Students from Universities B and G had a higher level of knowledge than students from Universities D and E. Senior students scored significantly higher than sophomores (*p* < 0.001) and juniors (*p* = 0.037). Female participants scored higher than male participants (*t* = −3.09, *p* = 0.002; Table 3).

**Table 3:** Results of the tests of associations between the Saudi nursing students' COVID-19 knowledge and demographic variables and perceptions related to COVID-19 *(n* = 1,226).

**Demographic variables and perceptions related to COVID-19**	**Saudi nursing students' COVID-19 knowledge**		**Statistical test**	***p***
	**Mean**	**SD**		
Age in years			*r* = 0.05	0.074
University^a^				
University A	9.82	1.53	*F* = 6.19	<0.001^***^
University B	10.18	1.51		
University C	9.84	1.34		
University D	9.33	1.99		
University E	9.65	1.75		
University F	9.71	1.55		
University G	10.13	1.47		
Gender				
Male	9.62	1.63	*t* = −3.09	0.002^**^
Female	9.94	1.60		
Year of study^b^				
2nd year	9.67	1.72	*F* = 5.67	0.001^**^
3rd year	9.81	1.71		
4th year	10.17	1.28		
Internship year	9.89	1.55		
Learned about coronavirus in any nursing course				
No	9.89	1.63	*t* = 1.78	0.139
Yes	9.74	1.58		
Family member working in a healthcare facility				
No	9.86	1.67	*t* = 0.21	0.837
Yes	9.84	1.57		
Perceived knowledge on COVID-19			*r* = 0.23	<0.001^***^
Perceived knowledge on COVID-19 prevention			*r* = 0.27	<0.001^***^

a *University B > Universities D (p < 0.001) and E (p = 0.008), University G > Universities D (p < 0.001) and E (p = 0.037)*;

b *4^th^ year > 2^nd^ year (p < 0.001) and 3^rd^ year (p = 0.037)*.

* *Significant at 0.05*,

** *Significant at 0.01*,

*** *Significant at 0.001*.

### COVID-19 Preventive Behavior and Associated Factors

The majority of the students performed 11 of the 13 preventive practices identified in the survey. Following the rules set by the government received the highest percentage of the “always” response (77.4%), followed by “throwing used tissues in trash cans” (75.4%), “avoiding close contact with sick people” (72.6%), “washing hands with soap and water for a minimum of 20 s after going to a public place” (72.3%), and “covering the mouth and nose with tissue when coughing or sneezing or using the inside of the elbow” (70.1%). Two preventive practices, namely, “washing hands with soap and water for at least 20 s after blowing my nose, coughing, or sneezing” (39.2%) and “daily cleaning and disinfecting frequently touched surfaces” (41.6%) were not always performed by the majority of the respondents (Table 4).

**Table 4:** Results of the descriptive analyses on the Saudi nursing students' preventive behaviors (*n* = 1,226).

**Items in the preventive behavior questionnaire**	**Never *n* (%)**	**Sometimes *n* (%)**	**Always *n* (%)**	**Mean (SD)**
1. I wash my hands with soap and water for at least 20 seconds after I have been in a public place	207 (16.9)	132 (10.8)	887 (72.3)	1.55 (0.76)
2. I wash my hands with soap and water for at least 20 second after blowing my nose, coughing, or sneezing	260 (21.2)	486 (39.6)	480 (39.2)	1.18 (0.76)
3. If soap and water are not readily available, I use a hand sanitizer that contains at least 60% alcohol	251 (20.5)	254 (20.7)	721 (58.8)	1.38 (0.80)
4. I avoid touching my eyes, nose and mouth with unwashed hands	241 (19.7)	301 (24.6)	684 (55.8)	1.36 (0.79)
5. I avoid close contact with people who are sick	232 (18.9)	104 (8.5)	890 (72.6)	1.54 (0.79)
6. I put distance between myself and other people	225 (18.4)	269 (21.9)	732 (59.7)	1.41 (0.78)
7. I stay at home if I'm sick, except to get medical care	213 (17.4)	186 (15.2)	827 (67.5)	1.50 (0.77)
8. I cover my mouth and nose with a tissue when I cough or sneeze or use the inside of my elbow	225 (18.4)	141 (11.5)	860 (70.1)	1.52 (0.79)
9. I throw used tissues in the trash	227 (18.5)	75 (6.1)	924 (75.4)	1.57 (0.78)
10. I wear facemask if I am sick	287 (23.4)	237 (19.3)	702 (57.3)	1.34 (0.83)
11. I clean and disinfect frequently touched surfaces daily	291 (23.7)	425 (34.7)	510 (41.6)	1.18 (0.79)
12. I clean surfaces that are dirty	248 (20.2)	200 (16.3)	778 (63.5)	1.43 (0.81)
13. I follow the rules implemented by the government during this COVID-19 outbreak	221 (18.0)	56 (4.6)	949 (77.4)	1.59 (0.78)

As shown in Table 5, one-way ANOVA revealed significant differences among preventive of the students when grouped according to university (*F* = 2.30, *p* = 0.033) and year of study (*F* = 5.40, *p* = 0.001). Students from University G and students in the internship year had better preventive behavior than students from University A (*p* < 0.05) and sophomores (*p* < 0.01), respectively. Female students (M = 18.96, SD = 8.49) had better preventive behavior than male students (M = 17.55, SD = 9.37, *t* = −2.43, *p* = 0.016). Weak positive correlations existed between preventive behavior scores and age (*r* = 0.09, *p* = 0.002) and between preventive behavior scores and perceived knowledge of COVID-19 (*r* = 0.08, *p* = 0.010).

**Table 5:** Results of the tests of associations between the Saudi nursing students' preventive behavior and demographic variables and COVID-19 knowledge (*n* = 1,226).

**Demographic variables and COVID-19 knowledge**	**Saudi nursing students' preventive behavior against COVID-19**		**Statistical test**	***p***
	**Mean**	**SD**		
Age in years			*r* = 0.09	0.002^**^
University^a^				
University A	17.38	9.46	*F* = 2.30	0.033^*^
University B	17.73	9.06		
University C	18.94	8.39		
University D	19.38	7.98		
University E	18.47	9.24		
University F	17.64	9.03		
University G	20.06	7.92		
Gender				
Male	17.55	9.37	*t* = −2.43	0.016^*^
Female	18.96	8.49		
Year of study^b^				
2nd year	17.55	8.95	*F* = 5.40	0.001^**^
3rd year	18.49	8.93		
4th year	19.12	8.77		
Internship year	20.49	7.61		
Learned about coronavirus in any nursing course				
No	18.72	8.58	*t* = 1.01	0.311
Yes	18.14	9.24		
Family member working in a healthcare facility				
No	18.48	8.66	*t* = −0.29	0.771
Yes	18.63	8.87		
Perceived knowledge on COVID-19			*r* = 0.08	0.010^*^
Perceived knowledge on COVID-19 prevention			*r* = 0.02	0.556
COVID-19 actual knowledge			*r* = 0.05	0.083

a *University G > University A (p = 0.050)*,

b *Internship year > 2^nd^ year (p = 0.001)*.

* *Significant at 0.05*,

** *Significant at 0.01*.

## Discussions

Our study focused on assessing the Saudi nursing students' perceptions, knowledge, and preventive behavior toward COVID-19. The findings of our study indicated that students primarily use social media to gather information on COVID-19, using various information sources, such as government agencies, the media, friends, and family (12). The current study's result is similar to that of Huynh and Nguyen (15), who reported that social media is the foremost source of COVID-19 information among healthcare workers in Vietnam. Another study reported that healthcare workers' sources for reliable information on COVID-19 are official government websites and social media (16). College students in Hong Kong held the highest level of trust toward health-related evidence provided on social media (17). On practical implication, the use of social media influences students' knowledge of the infection, such as the number of local and international incidents and knowledge of preventive measures. However, students should be responsible and focus on factual information they see on social media. This finding implies the need for nursing education to develop programs, such as educational and awareness campaigns aimed at guiding students to reliable student-centered sources of information about COVID-19. Nurse educators should assist students in selecting the right sources of information, provide student-centered resources, and correct misinformation. Additionally, this finding may help in modifying the contents of some courses (e.g., Infection Control in Nursing and Nursing Informatics) and enhance the means for acquiring dependable sources of information related to COVID-19. Future studies should focus on examining ways on how educators can assist students in carefully evaluating their sources of information.

Furthermore, the students were confident that the government and MOH are doing a good job in responding to the outbreak. The result of this study conforms with the result of the study conducted in China, where most of the respondents had positive attitudes toward the COVID-19 pandemic; that is, 90.8% thought that COVID-19 would be effectively controlled, and 97.1% were confident that China can overcome this outbreak (13). Saudi Arabia's MOH implemented several measures and guidelines designed for infection prevention and control, treatment, and public health considerations; healthcare workers were instructed to adhere to these measures strictly. The government of the country had implemented measures to safeguard the health of its citizens and residents and protect them from the disease. These measures aimed to mitigate the spread of the virus within the country (18). The residents of the majority of the regions in the kingdom were not allowed to leave their respective regions or move to another region, local and international flights were canceled, and curfew hours were strictly implemented. People believed that these strict preventive implementations can prevent and control the outbreak. Therefore, nursing education can play a role in disseminating information about the policies and protocols being implemented by the government to ensure their full awareness and compliance. In general, the information dissemination resources of universities, such as their social media accounts and university emails, can be utilized in communicating the government's efforts, guidelines, and protocols to the students.

Our findings revealed a good level of knowledge of COVID-19 among Saudi nursing students. The reported knowledge was slightly lower than the knowledge score reported among residents in China (13) and slightly higher than the score reported among US residents using the same questionnaire (19). The students' good knowledge of the disease revealed in our result may have been a result of the measures of the government, MOH, and Ministry of Education for improving the awareness of the residents on COVID-19 through information dissemination via social media and news outlets. The Ministry of Education had implemented measures to incorporate COVID-19-related topics in universities to ensure the adequate knowledge of university students. The effectiveness of such effort can be supported by our previous findings, which showed that the students had high confidence in the effort of the government and MOH in addressing the crisis and students highly rely on social media for COVID-19-related information. Our findings showed that students had the highest knowledge in the prevention and control aspect of COVID-19 and the lowest level of knowledge with regard to the transmission route, thereby corroborating the studies conducted in China (13) and US (19). This result can be attributed to the previous experience of Saudi Arabia with the MERS-Cov outbreak, which has similarities with the current pandemic in terms of preventive measures (20).

The level of COVID-19 knowledge among nursing students appeared to vary across different universities. Although each university has exerted efforts to increase the awareness of students of COVID-19, the strategies and frequencies of their educational campaigns may vary. For example, University B had successfully utilized the social media as an effective tool of information dissemination, raising the awareness about COVID-19 among its students (20). The university had implemented a framework for the use of social media to ensure the sustainable management of higher education during the pandemic (21). The university also developed and adopted a curriculum and training program that solely tackles information about the COVID-19 pandemic, preventive measures, and the significance of public health and research in managing the crisis (22). However, the survey was conducted after the government closed all schools, and this situation may have interfered with the information dissemination efforts of some universities. Differences in infection prevention knowledge and practices among Saudi students were also observed in previous studies, which attributed these differences to varying course contents, teaching methodologies, and other curricular activities (23, 24). Moreover, our findings indicated that female students had better knowledge than male students. This difference in COVID-19 knowledge between genders was also observed in China and the US, where female students scored higher than male students (13, 19). Gender differences on knowledge among Saudi nursing students had been documented in infection control prevention measures in previous studies (25, 26). The result also indicated that students' knowledge increased as they go higher in the academic ladder, indicating that senior students had a profound understanding of COVID-19-related information possibly because of their exposure to high-level learning and COVID-19-related information in their theoretical courses and hospital exposures. Thus, nursing education must ensure that student nurses in all levels have equal access to student-centered COVID-19 resources to prevent gaps on knowledge among students in different year levels. Moreover, no gap between perceived and actual knowledge on COVID-19 was observed in our study. This result was evidenced by the positive correlations between the students' perceived COVID-19 knowledge and their actual knowledge scores. That is, students who rated their knowledge high also received high scores in the actual COVID-19 knowledge survey.

On preventive behavior, among the variables measured, following the rules set by the government related to the COVID-19 crisis was the most observed preventive behavior. This behavior can be associated with the “Sharia law,” which refers to the correct Islamic behavior (27). Adherence, cooperation, and respect for the law is common to Saudi nationals, as further reflected by the good response of the students to the call of the government and health authorities to protect themselves and others. Furthermore, Saudi student nurses who abide by the imposed rules of the government regarding the prevention and control of COVID-19 is an indicator of their responsibility to Allah, the government, others, and oneself (27). As part of their responsibility to protect others, the proper disposal of waste, social distancing, hand washing, and proper coughing and sneezing etiquette in public are observed. The emphasis on the concept of infection control in all major nursing courses may have contributed to the good preventive behavior of the student nurses (28). However, the student nurses' low preventive practice of disinfecting surfaces highlighted vagueness among them regarding who is responsible for disinfection. This infection prevention practice received a lower emphasis than the other infection prevention practices, such as hand hygiene and using personal protective equipment during clinical practice. Moreover, the students may have observed during their clinical rotation that the disinfection of surroundings are often delegated to nursing attendants owing to the heavy workloads of staff nurses. These assumptions should be confirmed in future studies.

The preventive behavior of student nurses are linked to the university where they study and the year of study. These observed differences in preventive behavior may have been due to variations in the implementation of training programs of nursing schools in Saudi Arabia. The Saudi Licensing Exam blueprint specifically includes infection control as one of its sections (29). However, the absence of a unified nursing curriculum in the country may have contributed to the differences in preventive behavior among students of different universities (30). Moreover, every nursing school has its own program mission, goals, and learning outcomes, which have an impact on the acquisition and delivery of knowledge and the values, training, attitude, and desired level of competency or performance of student nurses. These differences between universities were also observed in the Saudi nursing student in terms of compliance with standard precautions in a previous study (28). Students on internships had better preventive behavior than those in other year levels, indicating that the practical learning experience in the clinical area of interns may have facilitated the development of their sense of risk perception. The development of the ability to sense and avoid hazards heightened their preventive behavior as a response to the health treatment of the novel coronavirus. Thus, the need for a unified nursing curriculum framework formulated by appropriate governing institutions, such as the Saudi Commission for Health Specialties and/or the Ministry of Education, is highly recommended.

This study used a convenience sampling method, which may have limited the generalizability of the findings. The cross-sectional nature of this research also hindered the examination of causal relationships between variables. The preventive behavioral patterns were self-reported, which may have been influenced by some social desirability bias. Nonetheless, our study is the first large-scale research that measured these variables in Saudi Arabia. The findings reflected the current perceptions, knowledge, and preventive behavior of nursing students as the COVID-19 crisis develops.

### Conclusions

Our study concludes that Saudi student nurses had good perceptions of their COVID-19 knowledge and its prevention, as well as positive perceptions on the government and MOH's effort in responding to the COVID-19 crisis. The students had good actual COVID-19 knowledge and preventive behavior against COVID-19. This study provides a basis for developing an educational campaign aimed at improving nursing students' knowledge of and preventive behavior against COVID-19. Having adequate knowledge and the correct preventive behavior against COVID-19 will ensure that nursing students are prepared to respond in future occurrences of similar public health crises. The findings of this study provide baseline information on the current state of the Saudi nursing students' perceptions, knowledge, and preventive behavior toward COVID-19 as the crisis is happening. The findings revealed some areas that should be focused on by nursing education, as well as MOH and other health agencies, for the purpose of ensuring that the population has adequate knowledge and correct preventive behavior. For example, knowledge of the disease's correct transmission routes should be a focus because this aspect received the lowest knowledge score. Some preventive measures should be emphasized, such as washing of hands after blowing your nose, coughing, or sneezing and the disinfection of surrounding areas, given that they were not frequently observed by the respondents. The findings may also guide the nursing profession with regard to its role in health promotion and disease prevention. Given that the main responsibilities of nurses include promoting health and preventing diseases, the findings may guide the creation of a health education program on COVID-19 for the improvement of the knowledge of the public, encouragement of their adoption of appropriate preventive behavior against the virus, and mitigation of the spread of the infection. Moreover, MOH and other health agencies can maximize the use of social media as an avenue for knowledge dissemination on COVID-19 because this platform is the primarily identified source of information. Contents shared or disseminated through social media should be checked for accuracy by relevant agencies to avoid misinformation and ensure that only factual information reaches the public.

## Data Availability Statement

The raw data supporting the conclusions of this article will be made available by the authors, without undue reservation.

## Ethics Statement

The studies involving human participants were reviewed and approved by Nursing Research Ethical Committee of King Abdulaziz University, Saudi Arabia. The patients/participants provided their written informed consent to participate in this study.

## Author Contributions

HMA contributed to conceptualization, design, study supervision, acquisition of data, interpretation of data, drafting and revision of the manuscript, approval of the final manuscript, and funding acquisition and agreed to be accountable for all the aspects of the work. NA, EB, and JB contributed equally to conceptualization, design, acquisition of data, data analysis, interpretation of data, drafting and revision of the manuscript, and approval of the final manuscript and agreed to be accountable for all the aspects of the work. HA, FA, RT, AA, HT, and EF contributed equally to the acquisition of data, interpretation of data, revision of the manuscript, and approval of the final version and agreed to be accountable for all the aspects of the work. JC contributed to conceptualization, design, study supervision, acquisition, analysis, interpretation of data, drafting and revision of the manuscript, and approval of the final manuscript and agreed to be accountable for all the aspects of the work. All authors contributed to the article and approved the submitted version.

## Conflict of Interest

The authors declare that the research was conducted in the absence of any commercial or financial relationships that could be construed as a potential conflict of interest.
